# Atypical diabetes arising from SHORT syndrome: a case report

**DOI:** 10.3389/fendo.2024.1467364

**Published:** 2024-12-13

**Authors:** Aili Wang, Miao Xu, Li Li, Jialin Li

**Affiliations:** ^1^ Department of Endocrinology and Metabolism, The First Affiliated Hospital of Ningbo University, Ningbo, Zhejiang, China; ^2^ Health Science Center, Ningbo University, Ningbo, Zhejiang, China

**Keywords:** SHORT syndrome, PIK3R1, whole-exome sequencing, insulin resistance, therapy

## Abstract

Short stature, joint hyperextension, ocular hypotension, Rieger abnormalities, and delayed tooth eruption (SHORT) syndrom is a rare primary autosomal dominant genetic disorder mainly caused by pathogenic loss-of-function variants in the phosphoinositide-3-kinase regulatory subunit 1 (PIK3R1) gene. We report the case of a Chinese adult female patient with SHORT syndrome, carrying a PIK3R1 gene variant (c.1945C > T), who developed abnormal glucose metabolism and severe postprandial insulin resistance over 9 years. Although there are currently no established treatment guidelines for insulin resistance in patients with SHORT syndrome, we implemented a comprehensive treatment plan, including lifestyle interventions, metformin, and voglibose for glucose control. After 6 months of continuous observation, the patient’s blood glucose levels and insulin resistance improved significantly. This case study provides useful insights for future treatment strategies.

## 1 Introduction

SHORT syndrome is an extremely rare genetic disorder primarily inherited in an autosomal dominant manner. Typical clinical manifestations include short stature, joint hyperextension, ocular hypotension, delayed tooth eruption, and Rieger abnormalities, characterized by underdevelopment of the iris and cornea, often accompanied by marked hypoplasia of the iris stroma, abnormal pupil positioning, and full-thickness defects of the iris (1, 2). Further understanding of the disease has revealed additional features in patients, such as intrauterine growth restriction, lipoatrophy, insulin resistance, and facial deformities (3). Fewer than 50 cases worldwide (4), the incidence, diagnosis, treatment, and mortality rates of the disease remain unclear. It is widely believed that the disease is primarily caused by a pathogenic loss-of-function variant in the phosphatidylinositol 3-kinase regulatory subunit 1 (PIK3R1) gene, located on the long arm of chromosome 5 (5q13.1) (1, 5). Study has shown that this variant in the PIK3R1 gene reduces the expression level of the p85α subunit, impacting downstream insulin signaling and predisposing individuals to insulin resistance and diabetes (4).

Here, we present the case of a Chinese adult female with a 9-year history of insulin resistance, who was ultimately diagnosed with SHORT syndrome due to a PIK3R1 gene variant (c.1945C > T). We reviewed relevant literature and implemented targeted treatment plans to provide new diagnostic and treatment strategies. This study was approved by the Research Ethics Committee of the First Affiliated Hospital of Ningbo University (Approval NO. 2024-144RS). Written informed consent was obtained from the patient.

## 2 Case presentation

The patient was a 33-year-old woman who had a spontaneous full-term vaginal delivery without birth trauma or asphyxia. Both parents were healthy and non-consanguineous, and no abnormalities were detected during prenatal checkups. At birth, the patient weighed 2000 g (< 3rd percentile), though her length was unknown. She exhibited delayed tooth eruption and speech development, with her first primary tooth emerging at 9 months and her ability to say “mom” and “dad” developing at 10 months. Throughout childhood, the patient consistently lagged in growth and development compared with their peers. At 5 years of age, her height was only 99.7 cm (−3 SD) and her weight 11.5 kg(< 3rd percentile). By age 9, her height was 116.5 cm (−3 SD) and her weight was 15.0 kg(< 3rd percentile). These persistent developmental delays concerned her parents, leading to multiple visits to local hospitals. Despite these delays, the patient’s intellectual development has remained normal. Her father was 167 cm tall and her mother was 160 cm tall. The patient had a younger brother, aged 29, who was 175 cm tall and weighed 60 kg.

The patient first presented to the endocrinology department of a local hospital at 24 years of age. Her test results revealed a fasting blood glucose (FBG) level of 5.48 mmol/L and 2-hour postprandial blood glucose level of 8.04 mmol/L. Notably, her postprandial 2-hour insulin level exceeded the upper detection limit (> 2152.5 pmol/L), while her postprandial 2-hour C-peptide level was 0.4 nmol/L. Her glycosylated hemoglobin (HbA1c) was 5.78%, indicating abnormal glucose tolerance and significant postprandial insulin resistance. Consequently, she was prescribed long-term voglibose monotherapy. In early December 2023, she was admitted to our endocrinology department for further evaluation due to persistently poor glycemic control lasting a week.

During hospitalization, a comprehensive evaluation was conducted, including a physical examination, laboratory tests, and screening for diabetes-related complications (**Tables 1**, **2**). The patient exhibited several notable characteristics, including short stature, joint hyperextension, distinctive facial features, delayed tooth eruption, an irregular menstrual cycle, and insulin resistance. Notably, despite elevated insulin levels, she did not exhibit other commonly associated features such as acanthosis nigricans, elevated plasma triglycerides, hepatomegaly, or hepatic steatosis (6). To investigate the potential genetic etiology, whole-exome sequencing was performed (**Supplementary Materials and Methods** in **Supplementary Data Sheet 1**). The results revealed a nucleotide variant at position 1945 in the PIK3R1, specifically a substitution of cytosine (C) with thymine (T) (c.1945C > T), leading to a heterozygous missense variant, resulting in an amino acid change from arginine to tryptophan at position 649 (p. Arg649Trp). This variant has been previously reported in the literature (7–12). The identified loss-of-function variants in the PIK3R1 are consistent with the pathogenesis of SHORT syndrome, which contrasts with the gain-of-function variants seen in APDS2, a condition characterized by immune dysregulation. Testing of the patient’s parents revealed no pathogenic variants at this site (**Supplementary Figure 1**). No features of APDS2, such as recurrent infections or autoimmune conditions, were observed in this patient. Based on these findings, the patient was diagnosed with SHORT syndrome.

**Table 1:** The comparison of typical features of SHORT syndrome with this patient.

Clinical features	SHORT syndrome	This patient
Typical characteristic	**S** (short stature)	**+**
	**H** (joint hyperextension)	**+**
	**O** (ocular hypotension)	–
	**R** (Rieger abnormalities)	**+**
	**T** (delayed tooth eruption)	**+**
Facial features	Triangle face	**+**
	Protruding forehead	**+**
	Hypoplastic or thin alae nasi	**+**
	Small chin	**+**
	Thin lips	**+**
	Low set ears	**+**
	Progeroid face	**+**
Other signs	Glaucoma	–
	Thin, wrinkled skin and visible veins	**+**
	Lipoatrophy	**+**
	Insulin resistance	**+**
	Glucose intolerance/diabetes mellitus	**+**
	Polycystic ovarian syndrome	**+**
	Intellectual deficiency	–
	Delayed speech development	**+**

**Table 2:** Results of laboratory tests, and diabetic complications of the patient.

Parameter	Value	Reference range
Height (cm)	147.50	–
Weight (kg)	37.50	–
BMI (kg/m^2^)	17.24	18.5-23.9
Lean mass (kg)	23.90	29.9-36.5
Fat mass (kg)	9.30	9.1-14.6
VFA (cm^2^)	35.3	<80
Total cholesterol (mmol/L)	4.20	3.00-5.70
HDL-c (mmol/L)	1.09	1.03-1.55
LDL-c (mmol/L)	2.92	1.89-4.21
Triglyceride (mmol/L)	1.47	0.00-1.70
Calcium (mmol/L)	2.19	2.11-2.52
Phosphorous (mmol/L)	1.25	0.85-1.51
25-hydroxyvitamin D3 (ng/ml)	14.5	>30
TSH (mIU/L)	1.97	0.56-5.91
Free T4 (pmol/L)	11.47	7.64-16.03
Total testosterone (nmol/L)	1.47	<2.60
Diabetic complication		
Diabetic retinopathy	–	–
Diabetic kidney disease	–	–
Diabetic peripheral neuropathy	–	–
Lower extremity arterial disease	–	–

BMI, body mass index; VFA, visceral fat area; HDL-c, high density lipoprotein-cholesterol; LDL-c, low density lipoprotein-cholesterol; TSH, thyroid stimulating hormone.

Initially, we prescribed metformin (500 mg, twice daily) as the treatment option. To evaluate the effectiveness of the treatment, continuous blood glucose monitoring was conducted on the fourth day after treatment initiation. However, one month later, the patient reported gastrointestinal complaints after taking metformin (500 mg, twice daily), and blood glucose monitoring showed persistently high levels after breakfast. In response, the glucose-lowering regimen was adjusted to metformin (250 mg, twice daily) and voglibose (0.2 mg with breakfast). To assess the effects of this adjustment further, the patient underwent repeated continuous glucose monitoring. Over a six-month follow-up period, we compared the glycemic profiles of the patients and summarized the results (**Tables 3**, **4**).

**Table 3:** Comparison of glucose metabolism indexes in three different periods.

Time	9 years ago	At admission	6 months after treatment
FBG (mmol/L)	5.48	6.08	5.52
Ins (0h) (pmol/L)	57.2	64.9	95.18
C-P (0h) (nmol/L)	0.40	0.84	0.90
PBG (2h) (mmol/L)	8.04	14.44	11.23
Ins (2h) (pmol/L)	>2152.5	>2100.21	1656.22
C-P (2h) (nmol/L)	2.33	5.98	7.30
HbA1c (%)	5.78	6.50	6.0
HOMA-IR	2.01	2.53	3.36

FBG, fasting plasma glucose; Ins (0h), fasting insulin; C-P (0h), Fasting C-peptide; PBG (2h), 2h postprandial blood glucose; Ins (2h), 2h postprandial insulin; C-P (2h), 2h postprandial C-peptide; HbA1c, glycosylated hemoglobin; HOMA-IR=FPG (mmol/L) * FINS (μU/ml)/22.5.

**Table 4:** The results of the patient’s continuous glucose monitoring.

Treatment	Project 1	Project 2	Project 3
Mean glucose (mmol/L)	7.13	6.54	6.43
TIR (%)	65.03	78.09	76.73
TAR (%)	33.57	18.10	18.10
TBR (%)	1.40	3.81	5.17
CV (%)	23.84	20.95	22.40

Project 1: Metformin alone (500mg, twice daily); Project 2: Combination of metformin (250mg, twice daily) and voglibose (0.2mg with breakfast); Project 3: 6 months after treatment. CGM, continuous glucose monitoring; TIR, time in range; TAR, time above range; TBR, time below range; CV, coefficient of variant.

## 3 Discussion

SHORT syndrome is a rare medical condition characterized by short stature, joint hyperextension, ocular hypotension, Rieger’s abnormalities, and delayed tooth eruption (2). However, it is important to note that not all patients will exhibit these five features. According to Magali et al., only about half of the patients show four or five of these classic symptoms (13). Additionally, some less typical characteristics, such as a triangular face, low-set ears, insulin resistance, and polycystic ovary syndrome, are also relatively common among patients (13). Our patient exhibited four of the five aforementioned classic features, as well as several atypical characteristics. The incomplete presentation of this syndrome can lead to it being overlooked during diagnosis, causing delays in timely identification and treatment. Therefore, when SHORT syndrome is suspected in clinical practice, it is essential to comprehensively consider the patient’s growth and developmental history along with relevant laboratory test results. Multidisciplinary collaboration, involving specialists in ophthalmology, dentistry, and endocrinology is also important for a comprehensive assessment of the illness. Genetic testing is a crucial diagnostic tool that can help establish a more accurate diagnosis.

Dyslipidemia is typically strongly associated with the onset of insulin resistance (IR). However, in SHORT syndrome, the underlying mechanism leading to IR is not a lipid metabolism disorder but rather a disruption in the insulin signaling pathway due to variants in the PIK3R1 gene (4). PIK3R1 encodes the regulatory subunit p85α of phosphoinositide 3-kinase (PI3K), which plays a crucial role in insulin sensitivity. It forms a complex, PI3K p85-p110, with the catalytic subunit p110α by binding and stabilizing it, promoting the conversion of phosphatidylinositol diphosphate (PIP2) to phosphatidylinositol triphosphate (PIP3). This process activates the protein kinase B (AKT) pathway, a critical mediator of insulin metabolic effects, including glucose uptake and metabolism in tissues such as muscle and liver (14). In patients with PIK3R1 variants, the down-regulation of the p85α subunit impairs the PI3K-AKT pathway, leading to defective insulin signaling and varying degrees of IR (4, 15). The mechanism of insulin resistance differs substantially from that of type 1 and type 2 diabetes. PIK3R1 variants can also lead to APDS2, which is involved in immune dysregulation due to the overactivation of the PI3K pathway. This could theoretically contribute to insulin resistance (16). However, in our patient, there was no clear evidence of immune-related issues affecting glucose metabolism. The patient exhibited no lipid dysregulation, with fat mass and visceral fat area falling below the normal range, accompanied by a reduction in lean body mass. Lipodystrophy is defined as the complete or partial loss of body fat (17). This condition can be categorized into two primary types based on the underlying cause: genetic or acquired. The distribution of lost adipose tissue further classifies lipodystrophy into congenital generalized lipodystrophy (CGL), familial partial lipodystrophy (FPLD), acquired generalized lipodystrophy (AGL), and acquired partial lipodystrophy (APL). Lipoatrophy resulting from SHORT syndrome is a rare variant of FPLD (18), therefore, clinicians must be particularly attentive during diagnosis. Additionally, according to previous studies, 9 of 17 cases (53%) of individuals with c1945C > T variant, which aligns with the variant site observed in our patient, developed IR and/or DM (7–12). Among these cases, 66.7% were female, with a median age of 30 years, and most occurred in European and American countries (77.8%). However, in patients with SHORT syndrome who did not develop IR or DM, we speculate that this result may be related to age, sex, race, disease duration, and other factors.

In terms of treatment, individual symptoms and dietary habits must be tailored. Over the chronic course of the disease, the patient’s fasting blood glucose, fasting insulin, and glycated hemoglobin levels did not show significant deterioration, but instead exhibited a sustained state of impaired glucose tolerance and postprandial insulin resistance. This finding is inconsistent with previously reported findings. Current reports on insulin resistance caused by SHORT syndrome have mostly focused on fasting and/or overall resistance, whereas relatively little attention has been paid to postprandial resistance (11, 12, 19). In addition, given that dietary carbohydrates constitute a significant portion of the Chinese diet (20), clinical practitioners should pay more attention to monitoring postprandial blood glucose and insulin levels. Secondly, metformin reduces hepatic glucose output, increases peripheral glucose uptake, improves peripheral insulin resistance, and reduces blood glucose (21). Voglibose, classified as an α-glycosidase inhibitor, selectively inhibits the functions of α-glucosidase, pancreatic α-amylase, and lactase. This inhibition effectively prevents the intake and hydrolysis of sugar, leading to a significant reduction in postprandial blood glucose levels (21, 22). Because of these attributes, α-glycosidase inhibitors are widely used in China. We speculated that the combination of these two drugs might be effective in treating hyperglycemia and postprandial IR, although there is currently no generally accepted effective treatment for IR in patients with SHORT syndrome. After 6 months of treatment, we noted significant improvements in glucose and postprandial insulin levels, but a notable increase in fasting insulin levels. Previous research has indicated that metformin may partially inhibit the PI3K/MAPK signaling pathway, potentially exacerbate IR in individuals with SHORT syndrome (23, 24). Therefore, clinicians should exercise caution and be aware of this potential risk when considering metformin as the primary therapeutic option for improving insulin resistance in this atypical form of diabetes. Third, a high-carbohydrate diet can stimulate insulin secretion; therefore, the importance of dietary guidelines in conjunction with drug therapy should not be overlooked (25). It is internationally recognized that patients with diabetes should be encouraged to increase their daily dietary fiber intake. The recommended food sources include whole grains, pulses, vegetables, and whole fruits (26). Finally, despite the patient’s poor glycemic control and high insulin levels, no diabetes-related target organ damage was identified over the past nine years. This phenomenon may provide new insights into the prognosis of diabetes caused by the SHORT syndrome. Whether this patient will develop chronic complications similar to those seen in type 2 diabetes over the long term, and whether targeted treatments are needed to reduce the occurrence of future complications warrant further investigation.

The patient was proactive in managing her condition. Upon receiving her diagnosis of SHORT syndrome, she felt relieved to have a clearer understanding of the cause of her symptoms, which had gone unexplained for years. She recognizes that it is a rare condition, making management potentially challenging; however, she remains optimistic about her long-term prognosis. Following the initiation of metformin and voglibose therapy, the patient experienced an improvement in postprandial blood glucose levels, alleviating some of her concerns. However, she remained concerned about the possibility of future complications related to insulin resistance, particularly given the lack of information regarding the long-term prognosis of SHORT syndrome. Additionally, she expressed concerns about the potential impact of her condition on future fertility. At this stage, she decided to prioritize managing her current metabolic and health issues before making decisions regarding pregnancy or family planning. The patient was committed to ongoing monitoring and lifestyle adjustments to better manage her condition and prevent potential complications.

The greatest strength of this study lies in its integration of the patients’ glucose metabolism data over the past nine years, along with dynamic monitoring of blood glucose levels and long-term follow-up after initiating treatment with metformin combined with voglibose. Additionally, owing to the scarcity of data, no guidelines for optimal diabetes care in patients with SHORT syndrome have been established. This study may provide useful insights into new treatment directions. However, this study has several limitations. First, owing to the lack of original medical records, we were unable to provide detailed information regarding her birth parameters, such as the Apgar score and birth length. Although we collected as much relevant information as possible, certain details from the perinatal period remained unavailable. Second, our assessment of IR status was based on HOMA-IR values and insulin release tests rather than the hyperinsulinemic-euglycemic clamp (HEC). Third, self-reporting may have led to recall bias. Finally, more time and research are required to comprehensively and accurately understand the prognosis of the disease.

## 4 Conclusion

In conclusion, we identified a heterozygous missense variant in PIK3R1 (c.1945C > T (p. Arg649Trp) in an adult Chinese female. The patient presented with the characteristic symptoms of SHORT syndrome, along with abnormal glucose metabolism and severe postprandial insulin resistance. A 6-month lifestyle intervention regimen combined with metformin and voglibose resulted in good glycemic control and marked remission of insulin resistance. Further studies are needed to explore the therapeutic utility and potential complications associated with SHORT syndrome.

## Data Availability

The original contributions presented in the study are included in the article/**Supplementary Material**. Further inquiries can be directed to the corresponding author.
