# Supplementary material for: Atypical diabetes arising from SHORT syndrome: a case report
Source: Front Endocrinol (Lausanne). 2024 Dec 13;15:1467364. doi: 10.3389/fendo.2024.1467364 (PMC11671247; doi:10.3389/fendo.2024.1467364)
Supplement: Supplementary file 1 [file DataSheet1.doc]

***Supplementary Material***

**Case Report: Atypical diabetes arising from SHORT syndrome: a case report**

**S1. Materials and Methods:**

Genomic DNA (gDNA) was extracted from the peripheral blood of patient following standard protocols. Whole exome sequencing (WES) using next-generation sequencing (NGS) technology was performed. This method targeted approximately 20,000 genes, including exons and adjacent splice sites (around 20 bp), as well as the entire mitochondrial genome. Sequencing was performed using a NovaSeq 6000 system (Illumina, USA). The reference genome standard used for this analysis was GRCh38/hg38. Sequencing data was analyzed using Genome Analysis Toolkit (GATK) for variant calling and Variant Effect Predictor (VEP) for variant annotation to identify pathogenic variants linked to hereditary diseases and assess genetic risks. Sanger sequencing was employed to confirm the identified variants. Familial variants were identified by Sanger sequencing in the proband’s parents. Data interpretation followed the guidelines of the American College of Medical Genetics and Genomics (ACMG), classifying the variants into five categories: benign, likely benign, unknown significance, likely pathogenic, and pathogenic [1].

**Reference:**

1. Richards S, Aziz N, Bale S, Bick D, Das S, Gastier-Foster J, et al. Standards and guidelines for the interpretation of sequence variants: a joint consensus recommendation of the American College of Medical Genetics and Genomics and the Association for Molecular Pathology. Genet Med. 2015 May; 17(5):405-24.

**S2. Figure 1:**


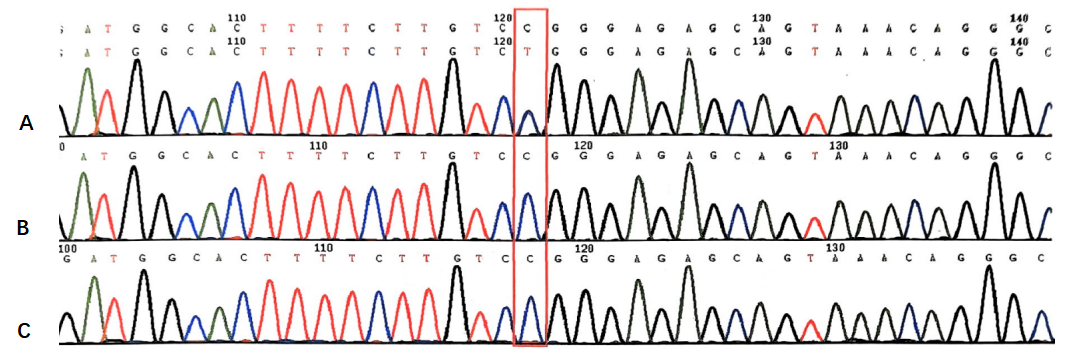


**Figure 1: The sequencing chromatogram of the variant**

A: whole exome sequencing showed a heterozygous missense variant of the PIK3R1 gene: c.1945C > T (p. Arg649Trp) in the patient. B: The sequencing chromatogram of patient's mother. C: The sequencing chromatogram of patient's father.
